# Clinical Lectures on Some of the Principal Diseases of the Eye

**Published:** 1855-11

**Authors:** Isidor Gluck

**Affiliations:** Chief Surgeon to the Hungarian (Vilmos) Hussars, and to various Hospitals during the late war in Hungary; Cor. Fellow Med. Soc. of London, etc.


// BUFFALO MEDICAL JOURNAL

AND

MONTHLY REVIEW.

VOL. 11. NOVEMBER, 1855. NO. 6.

### ORIGINAL COMMUNICATIONS.

ART. I.—*Clinical Lectures on some of the Principal Diseases of the Eye.* Delivered before the Class of the New York Medical College. By ISIDOR GLUCK, M. D., Chief Surgeon to the Hungarian (Vilmos) Hussars, and to various Hospitals during the late war in Hungary; Cor. Fellow Med. Soc. of London, etc.

(Continued from page 210.)

GENTLEMEN,—You see here the satisfactory result of the operation performed twelve days ago. Whereas before the operation the patient was annoyed by the blue and incorrect sight caused by the opacity in the right eye, he can now distinguish objects correctly with the right as well as the left one, and perceives, as you hear him say, no difference in the sight of both eyes. This marked improvement, calculated as it was before the operation, gives you an instance how the optical equality of the cornea may be restored by the operation of *abrasion;* although the opacity existed for many years, and the elements constituting it always underwent an exchange, like those of the normal part of the cornea, and thus the stationary appearance was preserved, and it shows you at the same time how little reäction follows the operation, so that the subsequent inflammatory process may be restricted to a small extent, and is easily controlled; while remedies hitherto applied for inducing absorption act on the whole extent of the cornea mechanically or chemically, thus jeopardising often the transparent part of the cornea, and thus adding to the optical inequality, by rendering the cornea more opaque in extent and depth.

The principle on which you perform this operation, is entirely different from that of cutting off slices in order to make the part thus transparent; successful cases of this kind are also recorded, and some thus operated upon even retained the transparency of the cornea, still it happens only too often that the opaque cornea made transparent by thinning it, will subsequently turn again optically unequal, and thus frustrate your exertion and render your tantalized patient more miserable than he felt before the attempt of restoring his sight was made.

It would be difficult to state to what an extent the abrasion in general should be performed, as the local deposit varies so much, not only according to its origin, but also to its nature; although it is pretty certain that in many if not in the most instances, the circumference of an opacity is easier resorbed than its center; but in those opacities in which many smaller ones are confluent and touch each other with their respective borders, so much as to present a uniform appearance, an interstitial resorption may take place, which may render the parts corresponding to the center of the opacity perhaps sooner transparent than the extreme border of the whole opacity.

In this instance I removed, as you observe, the lower part of the opacity, in order that the upper part of it corresponding to the pupil should be resorbed; the upper part of the opacity evidently presented a different aspect from the center; the exudation located there appeared much thinner, and to all appearance easier of transformation, losing itself, as it did, gradually between the healthy structured lamellæ of the cornea. Nature, by its intention of replacing the epithelium taken off from the center and lower portion of the opacity, used for that purpose the superfluous morbidly deposited exudation. The reparative process, in restoring the lost protecting epithelium, causes the nearest material capable of transformation, to enter into the completion of the normal constituent part.

I have purposely abstained from repeating this operation on this individual, presuming, as it turned out justly, that the *single* removal of the epithelial layers might be sufficient for the purpose of absorption of the transformable exudation.

Whenever a repetition of this operation is necessary, which frequently occurs, and is certainly preferable to the removal of thicker slices at once, it should not be performed until the excavation thus caused be filled up, or the epithelium covering it restored, in order to engage in the reparative process the surrounding parts, without eliciting a high degree of inflammation.

In the young lady, as some of you witnessed, I removed twice the superficial layers of the cornea, and will probably have to repeat the same operation several times in order that a resorbtion, of the desired extent, should take place; my expectations, also, for the restoration of good sight are, for many reasons, by no means as sanguine as they were in the present instance. I nevertheless entertain the hope, and even firmly believe, to succeed in it eventually.

The removal of the local deposit depends upon many circumstances, general as well as local: tender age is more favorable to it than the advanced; the constitution, if healthy, is more calculated of permitting an adequately speedy absorbtion than the cachectic one.

The capacity of transformation, being dependent upon local and general circumstances, will be facilitated if the product be more transmutable, on account of its diminished density, or in general will be more or less dependent upon the state of consistency of the product; provided the process of absorbtion is adequate to remove the mass softened, and thus ready to be carried off. The process of resorbtion will be most influenced by the nerves regulating the nutritive process in the eye. The disturbance of several physiological organic processes, constitute the character and nature of the hence resulting opacity, and may be differently modified by the numberless variations and possible combinations of such disturbances, creating and causing transformations into heterogeneous unserviceable masses, instead of being anatomically assimilated to serviceable and optically equal elements forming parts permeable to light. Although the process is one of a marked nature, based on laws conflicting with each other, the disturbed harmony may be, and often is, influenced by *accidental* external or internal circumstances, which decide upon the form and nature of such a morbid deposit *on* or *in* the cornea, or in any other part of the body, where the new formation depends upon the impeded physiological process, admitting an influence from within or without.

In inflammation, an excessive action may yield, by resolution, to proper treatment and to the efforts of nature, but if allowed to run its course, the issue will be new deposit, and hence resulting opacity or destruction of the parts healthy before the operation.

An interesting circumstance worthy of remark, is, that in individuals, although otherwise apparently healthy, inflammation of the eye will sometimes elicit symptoms characteristic of a latent disease which may not only essentially interfere with the reëstablishment of the sight, but the exudation may materially differ in aspect from products deposited in order to replace lost substances.

The exciting cause must be controlled and checked in its effect, and carefully watched throughout.

If I introduce to you now, two children afflicted with one and the same eye disease, it is, gentlemen, less from the intention of delivering us at once from the plaintive noise of both, but more from the fact that a simultaneous aspect of both will enable me to point out to you the different forms of one and the same eye disease, the possible result of which may be similar in its effect to the eye disease of the patient just discharged.

Clean and neat as the face of the *girl* looks, so ragged and sore appears that of the *boy*, covered on the chin and left cheek with an eruption half red and moist in its upper part, and half brown and dry in its lower part, denoting thus the different periods of apparition and evolution of the herpetic exanthem in the various spots of the face.

Both turn *from* the light, catch a glimpse and bury themselves in their respective mothers, perhaps not to show their faces again, as it often happens when children will not, or cannot listen to persuasion. The boy opens readily the right eye, but contracts the left orbicular muscle so far as to preclude the possibility of gaining a view of the left one. While the obstinate boy refuses to be persuaded to open the eye in the subdued light produced by shading it, the willing girl, one year younger (the boy being three years old) tries very hard to open either, but fails in her attempt of doing so, evincing excruciating pains by it; the tumefied heavy eyelids on the ciliary margin are richly covered with meibomian and ciliary glandular, partly hardened and crusty secretions, sticking together the eyelashes in bundles.

Both mothers, although living in different parts of the city, answer, as you hear, affirmatively the question, "whether the children suffered from measles?" The boy did so six months ago; while the girl had the measles about nine weeks ago; in both the eye disease occurred after, as they say, the measles left them. The boy, a French barber's son, sleeps in a basement not very lofty, his mother says, admitting but sparingly light and air; the little girl plays and sleeps together with several other children (all of them suffering more or less from sore eyes) in a basement, which, although situated in a healthy part of the town, in our neighborhood here, is neither large nor lofty, and only accessible by a smoky kitchen.

I now lift the upper and depress simultaneously the lower eyelid, in order to give you an opportunity of taking a glance at the left eyeball in the boy.

A rapid examination will be necessary, on account of the great intolerance of light. You observe vessels coming from different parts of the folds of the conjunctiva across the margin of the cornea where they terminate in the neighborhood of vesicles around which the cornea is dull; whilst you observe in the right eye of the girl but a few vessels, and none besides the bundle coming from the internal angle toward the border of the cornea, and no vesicles, but dullness, with excavations. You observe in the left one numerous vessels running to vesicles directly on the margin of the cornea, scattered on the upper part of the circle. The appearance is a characteristic one, although somewhat modified in form. This affection is called by Stellwag *Herpes cornea.*

Herpes in general is a disease of an acute and typic character simulating, by frequent successions of the product generated thereby, a chronic character; it originates with stinging and burning pain in the peripheral end of a *sensitive* nerve branch, and only subsequently becomes objectively perceptible by the appearance of solitary or of groupose conglomerated exudated products, localized on the extreme ends of a sensitive nerve branch, on the surface of the cutis or mucous membrane, and presenting themselves according to their character, sometimes as vesicles filled with serum as you see it on the chin of this boy; sometimes as nodules, without ever appearing in the stroma of a follicle.

All those symptoms you meet with in that form of ophthalmia which* Stellwag denotes with *Herpes cornea.* The modifications under which it appears are merely the consequences of the circumstances under which a deposit forms and exists on the conjunctiva and cornea.

A more or less violent burning or stinging *pain*, together with *intolerance of light*, precedes the eruption on the cornea. The cornea appears brighter than in its normal condition; the secretion of tears and the epithelial desquamation of the conjunctiva is increased. After a day or two, sometimes later, arise on the limbus conjunctivalis or on the corneal surface itself, *one*, or *several* vesicles, or grayish nodules of poppy seed or millet size. The exudation, however, may not establish itself to that extent, and a jellylike equable or nebulous opacity may form itself. The nodules may appear as *solitary*

---

* Mackenzie phlyctenular ophth.
(Hasner) Keratitis exanthematica.
Ophthalmia scrophulosa (conjunctivitis scrophulosa.
Ophthalmia pustularis.
(Hancock) Ophth. intermittens.

on one or another point of the limbus conjunctivalis, or may appear simultaneously grouped together on a portion of the circle formed by the limbus conjunctivalis around the cornea itself. The vesicles contain at first a limpid fluid, which appears curdy and dull from the change in the raised epithelium; but soon the fluid turns turbid-grey or whitish-grey, which changes and becomes yellowish pus-like, while at the same time a narrow halo is formed (by the dullness of the epithelial portion and of that part of the cornea that surrounds the base, and by deposit of a jelly-like gradually inspissating exudation) on which halo the vesicle is situated prominently. If several groups exist, the confluence of their several bases produces a common basis on which sometimes arises and distributes a net rich of vessels, in the loops of which the vesicles elevate themselves and are conspicuous.

If instead of vesicles, *nodules* appear at first, greyish-white elevations are visible, changing to white or yellowish white, and thus increasing in bulk protrude more, forming small cones situate with their base on the cornea or like plugs entering deeper in the cornea, and are surrounded by a greyish transparent halo of an opaque epithelium, and by an opaque exudation in which new vessels anastomozing, surround the cones on their base and now and then appear also on their surface. In those instances in which vesicles or nodules are not developed, herpes cornealis appears under the form of *a superficial vascular keratitis.*

The symptoms associated with the eruption are as manifold as the exanthem itself. They are partly of a nervous character, and are produced by a disturbance of the circulation in the parts of the eye carrying blood.

The disease beginning with burning and stinging pain, together with intolerance of light and its constant accompaniment of palpebral spasm and copious secretion of tears, shows itself under different degrees of the above symptoms. In many instances pain abates and intolerance of light diminishes; as soon as the peculiar efflorescence forms itself, the exudation goes through its metamorphoses without further trouble to the patient. Mostly, however, the pain and intolerance of light exists (as is the case when the eruption of the vesicles and nodules are protracted) for a long time, and the disease thus simulates *a vascular superficial keratitis.* With the appearance of the peculiar efflorescence the nervous excitement subsides. The pain and the intolerance of light being caused by one and the same source, both appear, increase, decrease and subside simultaneously.

*Pain and intolerance of light* denote one and the same symptom: the first by want of objective light, the last by the effect of light. They vary in the most different degrees from slight pain during the brightest light, to the intolerance even of the smallest quantity, and the most excruciating pain may be felt even by total absence of light, so much that the patient locks out in the obscure room for the darkest spot, and presses with force his eyes against an object, thus to find some relief. Pain and intolerance of light are always remitting, sometimes perfectly intermitting; without that no regularity could be traced in the intermission and the exacerbation. Mostly, however, they show a quotidian type, by being more exacerbating and violent in the morning; it occurs, however, that the symptoms intermit for a few days and thus present a tertian or quartan type, or even one of eight days or a fortnight. The disturbance in the circulation of the conjunctiva and in the epithelial texture are often characteristic. Soon after pain and intolerance of light sets in, before the efflorescence is yet visible, new vessels develop themselves in the conjunctiva, coming toward the margin of the cornea from the folds of the conjunctiva passing from the lid to the eyeball, the vessels multiplying and extending themselves from a bundle assuming the form of a strip, or oftener, as you see it in this boy, of a fan, the base of which is situated in the folds, and its apex on the margin of the cornea. If the efflorescence is localized on the limbus conjunctivalis, its base and the apex of the vascular bundle constantly meet, and the efflorescence develops itself in the central end of the vascular bundle. If, however, the vesicle or nodule is localized on the surface of the cornea, the bunch of vessels appears chopped off and a bridge of a jelly-like opaque corneal substance leads to the efflorescence. Soon after, a few hours after the appearance of such an opacity, vessels develop themselves, exist and appear like continuations of conjunctival vessels; the bunch of vessels seems to extend over the corneal surface on to the exudation. Frequently, however, not *one* bundle of vessels presents itself, but the conjunctiva of the eyeball is in its whole extent covered by enlarged vessels and bundles taking a centripetal direction, and then usually several efflorescences are visible on different parts of the cornea, as you see it in the girl. At the same time the conjunctiva is evidently infiltrated with a serous fluid, and a little swollen, not unfrequently similar small vesicles are visible which have been formed by infiltration of lymph and subsequent raising of the epithelium; the conjunctiva of the palpebra almost always shows catarrhal symptoms. The follicles are swollen, presenting, as here, the appearances of a slight partial trachoma, and the secretion formed in the conjunctiva is morbidly altered. It consists chiefly of mucus mixed with tears, which, under the microscope, presents itself as a structureless viscid, transparent, thin, and light granulated mass. It contains much meibomian fat, in form of larger and smaller coagula, and epithelium of recent formation. Pus cells and nuclei are met with only when catarrh attains a higher evolution.

The conjunctiva covering the sclerotic coat is congested to a smaller or larger extent, and swollen from infiltrated serous, often also jelly-like, and more consistent masses. In a partial congestion the pink net of vessels exists on those spots on which the superficial conjunctival vessels take their course, and therefore but a strip of the subconjunctival texture is altered. In the region of the sclerotico-corneal junction the congestion is the greatest, a portion of the circle, a segment of the sclerotical edge, appears in form of an elevated pink wall, the middle of which the vascular bundle crosses. From its central margin proceed small vessels branching off dichotomically forming a smaller or larger circle around the cornea, and at last inosculate in the limbus conjunctivalis, in order to communicate with the vessels of the corneal opacity. The injection and swelling of the episclerotic texture is more constant than even that of the conjunctiva itself. The former is never absent when the latter exists; often, however, in the conjunctiva are met with only single branches, or none at all; while in the subconjunctival texture impediments of circulation are evident.

The congestion and infiltration of the conjunctiva, and of its subjacent cellular texture, is not always proportionate to the nervous excitement. Pain and intolerance of light may vary exceedingly, and the congestive symptoms may scarcely be apparent; and often the congestion and injection of the episclerotic texture is enormous, while the nervous symptoms subside with the appearance of the efflorescence. But if both nervous excitement, and impediment of circulation or congestion are associated simultaneously, the mutual causal nexus must be recognized, as with the exacerbation and paroxysm of pain and photophoby constantly appears an increase of redness produced by the injection of the vessels. There are, however, instances in which the exanthem of the cornea is neither accompanied by nervous symptoms, nor by impediments of circulation in the blood-carrying organs, the pain disappears with the eruption of the efflorescence, and the latter stands isolated surrounded by entirely normal corneal parenchym, and is the only symptom of an existing morbid process in the cornea. Each efflorescence runs typically. The cycle of the specific process causing it terminates within eight days. The subsequent metamorphoses after that time are no longer typic, they are the same which take place under existing circumstances in other products of a morbid process, and it depends upon the constitution of the exudation generated by the specific morbid process, and upon the circumstances under which the exudation is placed during and after the course of the specific process, and thus is influenced the form and appearance of this disease. We meet, therefore, with herpes cornealis assuming a form and a course not common to a herpes of the cutis, but finding its analogue in the center of localization on the mucous membrane.

The herpetic exudation is, according to Stellwag, never deposited in the stroma of a follicle, but is localized in the peripheric end of a nerve tubule, and appears, therefore, on the skin and mucous membrane in the region of a papilla. The herpes cornealis equally localizes its product on the surface of the cornea where it appears conglomerated, and raises the epithelium in the form of vesicles and nodules. The subtility of the corneal epithelium causes deviations in the specific form of the herpetic exanthem, chiefly if the exudation is fluid, and the epithelium raised to a vesicle. The herpetic vesicle form a single cavity, the increase of deposit in it multiplies the pressure which each particle of the raised epithelium has to sustain, and a laceration is followed by an elimination of all the contents of the efflorescence, and thus a total change of form is produced. Sometimes a vesicle will burst at the outset of an exudation before it terminated, and before a metamorphosis of the exuded mass has taken place. A shallow excavation results from it, which is surrounded by epithelial *rags;* and if the exudation was situated on the limbus conjunctivalis, it shows a gelatinous base; whereas it presents a limpid base if situated on the surface of the cornea. The exanthem appears, under such circumstance, like a slight abrasion of the cornea—like what is called an ulcer of *resorbtion.* It is nothing but a destroyed herpetic efflorescence with a watery product. The localization of the peculiar herpetic product on this abrasion continues till the completion of the cycle peculiar to herpes; the corneal herpes is, in its course, similar to an ulcer of resorbtion. In other instances the exudation is, from its beginning, a more plastic one and more prone to differentiation or separation, or the vesicle bursts later, after the originally rough serum resembling exudation has already separated its plastic elements by coagulation. These localize like all centers of exudation on the walls of the cavity, and after destruction of the efflorescence the base of the ulcer is found covered by a more or less dense stratum of a turbid viscid mass, which gradually increases in thickness by new deposits, if the process is not finished yet, and thus after the completion of the typic course, appears like a lardy deposit on the place of the efflorescence. It rarely happens that the exudation can have time for being transformed into pus before the vesicle ruptures. If the rupture ensues, the base is covered by a pus-like exudation; herpes appears under the form of a small ulcer. In certain cases the vesicle does not burst, the contents metamorphose further and inspissate through the exosmose of the fluid parts, the vesicle bursts and appears as a nodule. The nodule has, in consequence of the greater plasticity of the product forming it, a greater duration, the further metamorphoses appear only later after the typic process terminated. The same is applicable to herpes appearing as keratitis with formation of vessels. Thus the herpes cornealis terminates the cycle of its specific metamorphoses under various forms, and enters in the order of non-specific forms, the course of which is a most different one, as well in relation to time as to its consequent stages.

The herpes cornealis is liable to reäppear, which modifies the acute typic course of the corneal herpes, and protracts it indefinitely. Not unfrequently, in a short time, one vesicle or nodule appears after the other, or a specific efflorescence varies with a diffuse deposit, by which it appears as if the efflorescence would wander from one place to another, and would assume a chronic course. But those are but successions, exact observations show that the place of the efflorescence is *unalterable*, and its course a *typic* one, but its terminations *vary* exceedingly according to circumstances.

Usually the first eruption is a rich one, a whole group of efflorescence develops itself on a more or less extended common ground. The herpetic eruption is situated partly on the cornea or on the corresponding portion of the conjunctival limbus and the conjunctiva. It consists partly of vesicles, partly of nodules, which undergo changes peculiar to them, and are connected with each other by a diffused, at first jelly-like, and then vasculous, even granulating exudation. The mass becomes increased by steady successions, a large portion of the cornea appears pannous, and subsequently it is changed to a tendon-like texture, on which a number of vessels run, and new vesicles, nodules of different standing, and ulcers are united with each other. The conjunctival limbus becomes irrecognizable, the exudative mass of the cornea is in immediate connection with the entirely metamorphosed adjacent part of the conjunctiva and subconjunctival texture; the limit between both disappears. The last textures are swelled by imbedded exudation; often they are several lines high, elated over the surface of the remainder of the conjunctiva, and form a kidney-like swelling, the hilus of which enters in the altered corneal texture, while the convex irregular ragged margin looks outward and often terminates abruptly in the normal conjunctival texture. This limbus occupies a greater or smaller circle of the cornea, is darkened in consequence of the development of numberless vessels, and is covered with a number of nodules of millet or canabis seed size, which sometimes are sago-like, transparent jelly; sometimes whitish-gray-like cartilage; now and then they appear yellowish pus-like, and prominent; and not unfrequently they are mixed together with watery, turbid, and often with vesicles containing pus, and more or less extensive ulcers of the most different form. Out of the convexity of that limbus run several bundles of thick widely dilated vessels, in a centrifugal direction to the corresponding portion of the transitory fold in which they inosculate and disappear.

The metamorphoses of that portion of the exudate which is used for the formation of the common ground of the efflorescences, are always constant ones. In the cornea it develops itself in the way of splitting into fibres, partly in the manner of cell formation in the conjunctiva, however, and its subjacent cellular tissue exclusively as cells to higher degrees of organization.

Disturbance of sensibility is one of the first and one of the chief symptoms of herpes cornea; the characteristic burning and lancinating pain appears before the disturbance of the circulation, and before the exudation takes place, and disappears often with the exudation; and if the pain is not persisting it is in no way proportionate to the intensity of the injection of the vessels or to the exudated mass, and frequently the pain is excruciating while the disturbance of the circulation is slight, and no exudation of any consequence had been deposited. An alteration of the nervous state can, therefore, in this instance, not be the consequence of a morbid alteration of the blood circulation, and there exists no connection with the appearances of the exudation; the *pain and intolerance of light must, therefore, be regarded* as the expression of a primary morbidity of single branches of the ciliary nerves, the more so as they retain this character, in most cases, by the obvious typic intermissions. On the contrary, however, there exists many circumstances tending to prove that the disturbance of circulation and the exudation is attributable to a nervous affection. We are forced to that conclusion even in such cases in which injection of the neighboring blood-carrying vessels is visible, as the exudation does not as usually, in inflammations, establish its center in the middle of the congested center, but almost always on its margin and extreme border, and mostly on places in which the most branches of nerves terminate, that is to say, in the limbus conjunctivilis.

The congestion and infiltration of the conjunctiva and of the subconjunctival texture is a frequent accompaniment, and is like the disturbances of circulation and œdemas occurring in the region of radiation of hypersthætic nerves, as met with in neuralgia frontalis or that of the face. The extent of the congestion and of the œdema is here proportionate to the number of the affected nerve tubes, and its seat is confined to the part in which the hypersthætic nerve radiates; in the same way may the congestion and infiltration of the cornea be attributable, in herpes, to the alteration of distinct branches of the nerves of the eye. The morbid alteration of the conjunctiva and its subjacent cellular texture may depend from the affection of those branches of the fifth nerve which radiate in the mentioned parts without touching the ophthalmic ganglion, a supposition which gains probability by the fact that the herpes in the eye, if accompanied by intense congestion and infiltration of the conjunctiva, and of the episclerotic texture often appears associated with herpetic efflorescences on the periphery of the frontal, lachrymal, and infraorbital nerve; the alteration causing herpes existing in a larger branch of the trigeminus. The branches of the fifth nerve running internally in the eyeball may be unaltered, and, therefore, pain and intolerance of light recede, and only an increased secretion of tears is observed in consequence of an irritation of the sensitive branches of the nerves in the orbit.

Cases in which no congestion of the conjunctiva is observable, and in which only a portion of the sclerotic margin is injected, are caused by an alteration of the *ciliary nerves* coming out of the *ciliary ganglion* piercing the sclerotic wall, and running between the sclerotic and choroide coats to the ciliary muscle, in order to distribute partly in the latter, partly in the iris and the cornea. These nerve twigs have naturally little or no influence over the conjunctiva and its subjacent cellular texture, their morbid alteration can therefore take place without visible redness, or at the most cause an injection and swelling only in the sclerotic margin. It is obvious, for that reason, that the efflorescences caused by those nerve twigs are always nearer to the center of the cornea and usually affect deeper the cornea; and it may be understood why the greatest intolerance of light is observed in cases in which the redness produced by congestion of the conjunctiva and of the episclerotical texture is small or does not exist at all.

The changable form of herpes cornealis finds, therefore, its explanation in the anatomical distribution of the affected nerve twigs; on the other hand the external objective symptoms of herpes are calculated to explain the great influence the nerves have on the vegetative life of the single organs, and chiefly to show the dependence of the exudations and new formations in the cornea from disturbances of the normal nervous impulse. Corneal herpes proves best that the ultimate cause of keratitis is to be looked for in disturbance of nervous activity, and that the same is to be considered as an exudation independent of disturbances of the circulation. The intimate connection existing between the nervous function and the exchange of elements in the cornea, is evident, not only from the dullness and softening of the cornea if the communication of the nerve or capacity of transmitting its nervous influence is impeded, but is also convincing from the appearance of keratitis and corneal ulcerations if the trigeminus is morbidly irritated.

### HERPES CORNEA

Is one of the most frequent diseases of the cornea; it is mostly caused by a morbid alteration of a *branch of a sensitive nerve;* young individuals, from two to seven years of age, are chiefly affected by this disease; easily irritable, spirited children, with great volubility, are mostly subject to it; it decreases in the development and severity of its nervous symptoms, the stronger the constitution and the more advanced the age.

Acute exanthematic diseases, as *variola*, *measles*, and *scarlatina*, mostly dispose the eye to the formation and development of herpes on it, where it appears frequently *after* those exanthematic diseases existed on other parts of the body. It is to be presumed that not only the general debility arising during that disease, and the *invitability* hence resulting; but also the intrinsic connection of the membranous coverings of the surface of the body, contributes to the establishment of herpes in the eye. The intimate relation in which the several symptoms are, by means of the sensitive nerves, accounts for the appearance of herpes in the eye as a *consecutive* disease. This is chiefly the case if the exanthem exists in the face, consequently in the region of radiation of the fifth nerve when herpetic efflorescences occur, not only in the mucous membrane and in the cornea of the eye, but also on the mucous membrane of the nose and mouth. The sympathetic irritation of the ophthalmic branch may be caused, not only by the inflammation producing the exanthem, but also by the influence of the sharp and often corroding secretion at the peripheral ends of the cutaneous nerves, a circumstance much favored by the *rare* use of water, which many avoid, out of fear to suppress the exanthem, and thus give opportunity for a chemical change of the product that then becomes *acid* and *rancid*, and the more easy is this process if salves and plasters are applied as local remedies. The sympathetic irritation is not only produced in neighboring trunks, often the body is covered with different efflorescences, although the original seat of the exanthem may have been restricted to a single circumscribed spot. These eruptions are entirely sufficient for keeping the patient, by their constant stinging, burning and pruritus, in a continued excitement. The patient scrapes day and night, his body is deprived of rest, and thus the nervous system becomes disposed to herpes, the more so as the vegetative life is also altered by it morbidly. An improper treatment with vesicants and setons, together with a repletion of the stomach with drugs of different kinds, will then easily contribute to the production of dyscrasia with consecutive grave diseases hence resulting.

The cases of herpes cornealis are more frequent in the spring and fall than at any other time, which may be attributed to the circumstance that the children, during winter restricted and confined to warm rooms, are, in the spring, brought in the open air and are exposed to many circumstances giving rise to the development of the disease, and after having been accustomed to the free open air, are again retained in the fall in the rooms, perhaps not over neat, many of them damp, dull and smoky abodes.

Every traumatic excitement, a foreign body impinged in the eye or between the lids, irritating substances, smoke, sharp and acid gases, may produce herpes, chiefly if it existed already before in the eye; indeed even a cool wind may, by a predisposition in the eye, produce herpes. If, in consequence of cold, catarrh develop itself in the mucous membrane of the nose and the eye, the herpes may reäppear in young individuals, as well as in adults. The frequency of a simultaneous eruption of herpes on the lid of the eye by suppressed transpirations, is striking.

It is the trigeminus that transplants the sensibility occurring during *dentition*, in children, to the eye, and thus causes the herpes so frequent under similar circumstances.

Alterations of single nerves in the eye transplant themselves easier on the other branches of the ophthalmic portion of the trigeminus, than from the rest of the branches of this nerve. We meet, therefore, frequently with herpes cornealis, as a complication of the most different inflammatory forms of the eye and its surrounding parts. It usually occurs together with the inflammation of the ciliary glands in the eyelids.

Herpes cornealis may leave opacities which are absorbed under favorable circumstances by nature, or by assistance of remedies, while under less favorable conditions it may leave opacities that may require the operation for abrasion.

*You recollect, gentlemen, the opacity in the left eye of the young lady of Morrisania. The sight of the young lady is now rapidly improving; the removal of the epithelium and of some of the subjacent tissue, was effected four times in five weeks. The young patient is not only capable of walking alone in the house, but finds her way, without apposition of the hand to the face: in the street a hood, advised for the purpose, is used. Although the excessive light in these hot days produces, of course, a great diminution of the

* Delivered six weeks later.

pupil, I had this morning the satisfaction to hear from her, joyfully relating that she had recognized a pin on the ground, which afterward she picked up. I held several small objects before her eye, which she recognized and could name correctly. I was not a little puzzled, as I found, a fortnight ago, that she could recognize objects even smaller than the largest letters of Jäger (No. 20), and still to my question, whether she could name the letters of No. 20, she answered, after considerable exertion of bringing the book variously nearer to or from the eye, "No." Even the assistance of the hood could not make her tell the letters, for the simple reason, which I learnt after a long trial, that she had forgotten her A B C's, having been blind for so many years. Now there is but a central speck, also diminishing in circumference; it will take a long time for its resorbtion, if it should take place at all.

However, the young lady can now distinguish perfectly, larger and smaller objects and learns, also, how to read. The middle sized letters of Jäger's numbers she easily recognizes; the exertion for reading the small letters would be an injurious effort and possibly might give rise to inconvenience if continued. I, therefore, advised the exclusive use of the larger letters until the accommodation of sight admits of the use of the smaller print. The young lady, satisfied with the success she now enjoys, not only presents a happier countenance, but shows, also, by seeing correctly, a marked improvement in her movements, which, in consequence of the exertion of seeing, were less graceful.

